# The Chemical Complexity of e-Cigarette Aerosols Compared With the Smoke From a Tobacco Burning Cigarette

**DOI:** 10.3389/fchem.2021.743060

**Published:** 2021-09-30

**Authors:** J. Margham, K. McAdam, A. Cunningham, A. Porter, S. Fiebelkorn, D. Mariner, H. Digard, C. Proctor

**Affiliations:** ^1^ Group Research and Development, British American Tobacco, Southampton, United Kingdom; ^2^ McAdam Scientific Ltd., Eastleigh, United Kingdom; ^3^ Independent Researcher, Montreal, QC, Canada; ^4^ Mariner Science Ltd., Salisbury, United Kingdom; ^5^ DoctorProctorScience Ltd., Ascot, United Kingdom

**Keywords:** e-cigarette, flavor, aerosol chemistry, targeted, untargeted

## Abstract

**Background:** As e-cigarette popularity has increased, there is growing evidence to suggest that while they are highly likely to be considerably less harmful than cigarettes, their use is not free of risk to the user. There is therefore an ongoing need to characterise the chemical composition of e-cigarette aerosols, as a starting point in characterising risks associated with their use. This study examined the chemical complexity of aerosols generated by an e-cigarette containing one unflavored and three flavored e-liquids. A combination of targeted and untargeted chemical analysis approaches was used to examine the number of compounds comprising the aerosol. Contributions of e-liquid flavors to aerosol complexity were investigated, and the sources of other aerosol constituents sought. Emissions of 98 aerosol toxicants were quantified and compared to those in smoke from a reference tobacco cigarette generated under two different smoking regimes.

**Results:** Combined untargeted and targeted aerosol analyses identified between 94 and 139 compounds in the flavored aerosols, compared with an estimated 72–79 in the unflavored aerosol. This is significantly less complex (by 1-2 orders of magnitude) than the reported composition of cigarette smoke. Combining both types of analysis identified 5–12 compounds over and above those found by untargeted analysis alone. Gravimetrically, 89–99% of the e-cigarette aerosol composition was composed of glycerol, propylene glycol, water and nicotine, and around 3% comprised other, more minor, constituents. Comparable data for the Ky3R4F reference tobacco cigarette pointed to 58–76% of cigarette smoke “tar” being composed of minor constituents. Levels of the targeted toxicants in the e-cigarette aerosols were significantly lower than those in cigarette smoke, with 68.5–>99% reductions under ISO 3308 puffing conditions and 88.4–>99% reductions under ISO 20778 (intense) conditions; reductions against the WHO TobReg 9 priority list were around 99%.

**Conclusion:** These analyses showed that the e-cigarette aerosols contain fewer compounds and at significantly lower concentrations than cigarette smoke. The chemical diversity of an e-cigarette aerosol is strongly impacted by the choice of e-liquid ingredients.

## Introduction

Since their emergence in the early 2000s, e-cigarettes have emerged as popular alternatives to tobacco cigarettes. Reviews of the e-cigarette science base suggest that while the absolute risks of vaping cannot yet be determined unambiguously, e-cigarette use appears to be associated with reduced exposure to many cigarette smoke toxicants (National Academies of Sciences, Engineering, and Medicine, Health and Medicine Division, Board on Population Health and Public Health Practice, Committee on the Review of the Health Effects of Electronic Nicotine Delivery Systems, 2018; PHE 2019]. However, e-cigarette use is not free from risk, with reports of adverse events in the pulmonary, oral, gastrointestinal and other bodily systems following exposure to e-cigarette aerosol (Seiler-Ramadas et al., 2020).

Historically, an established starting point in characterising the risks of using nicotine inhalation products has been the thorough chemical characterisation of the matrix inhaled by the user. For example, in the case of cigarettes, over 60 years of detailed scientific work undertaken to elucidate the chemical composition of both tobacco and smoke have highlighted their extreme chemical complexity at over 8,000 identified compounds in tobacco and over 6,500 identified compounds in cigarette smoke (Rodgman and Perfetti 2013). Clarity over the numbers, identities and concentrations of these compounds has enabled scientists, non-governmental agencies and regulators to compile priority lists of smoke constituents that are considered to contribute to the toxicity of cigarette smoke (Hoffmann and Hoffmann, 1988; Health Canada 1999a; Liu et al., 2011). These constituents have been referred to variously as “biologically active agents,” toxicants, and Harmful or Potentially Harmful Constituents (HPHC). In 2008 the WHO’s Tobacco Product Regulation (TobReg) working group proposed 9 toxicants for mandated lowering of levels in cigarette smoke (Burns et al., 2008). More recently, the US Food and Drug Administration (FDA 2012) published a list of 93 cigarette smoke HPHCs, with reporting requirements for a subset.

The growth in e-cigarette use as an alternative to cigarette smoking has led to significant efforts to understand the chemical composition of both e-liquids and e-cigarette aerosols. In contrast to the chemically diverse composition of tobacco, e-liquids are in principle compositionally simple, being composed of four main constituents: vegetable glycerol (VG), propylene glycol (PG), nicotine and water. However, there are also many thousands of flavored e-liquids available for sale, (Zhu et al., 2014; Krüsemann et al., 2021), whose flavor character is made up of synthetic flavor compounds, extracts of natural materials, or combinations of these. In addition to flavor compounds, minor components of ingredients and device materials, and potential reaction products of flavor compounds with major e-liquid components, and device materials, can extend the chemical complexity of the e-liquid. There is growing interest in the chemical composition of e-liquids, particularly the number, identities, quantities and toxicological impacts of flavorants used in them (Erythropel et al., 2020; Krüsemann et al., 2021; Omaiye et al., 2020).

E-cigarette aerosols are more chemically complex than e-liquids, due to formation of reaction and degradation products when the e-liquid is heated during aerosolisation. E-cigarette aerosol studies tend to use targeted HPHC analyses (National Academies of Sciences, Engineering, and Medicine, Health and Medicine Division, Board on Population Health and Public Health Practice, Committee on the Review of the Health Effects of Electronic Nicotine Delivery Systems, 2018), such as, carbonyls (Farsalinos and Gillman, 2018), metals (Williams et al., 2013) and major e-liquid component thermal decomposition products (Uchiyama et al., 2020). A few e-cigarette studies have targeted broader ranges of HPHC emissions, Lauterbach and Laugesen (2012), Tayyarah and Long 2014, Cunningham et al. (2020) with up to 150 measurands examined (Margham et al., 2016). These studies have shown considerable differences between e-cigarette aerosols and cigarette smoke, detecting fewer HPHCs in e-cigarette aerosols, and lower concentrations of those that are present. Such “targeted analyses” are powerfully informative in that they use analytical methods appropriate to the analyte being investigated, offer clear identification of the species present, and can quantify their concentration. However, a drawback of targeted analyses for specific, compounds (particularly cigarette smoke HPHCs) is that even the broadest study is unlikely to cover all of the constituents or toxicants present in e-cigarette aerosols. For example, a number of flavor-related chemicals of toxicological concern have been identified in e-cigarette aerosols, but were not prioritised in historic cigarette smoke toxicant lists, e.g., diacetyl (Allen et al., 2016; Vas et al., 2019), cinnamaldehyde (Behar et al., 2016), furfurals (Soussy et al., 2016), benzaldehyde (Kosmider et al., 2016), and vitamin E acetate (Boudi et al., 2019).

An alternative approach for examining the breadth of compounds present in a matrix is to conduct an “untargeted” analysis. Untargeted GC-MS approaches have shown greater capability for this type of analysis than their HPLC-MS counterparts. GC approaches use thermal conditions that are very consistent with the operating temperatures of e-cigarettes, suggesting analytical compatibility with the aerosol species present; also GC-MS libraries currently offer greater capability than HPLC-MS libraries. Two studies have reported successful application of untargeted GC-MS analysis to e-cigarette aerosols and have identified similar numbers of compounds. Using thermal desorption GC-MS (TD-GC-MS) Herrington and Myers (2015) identified 85 aerosol compounds. Using TD-GC-TOFMS Rawlinson et al. (2017) identified 33 compounds in an unflavored commercial e-cigarette product, and 69–87 compounds in flavored e-cigarettes. GC-MS approaches are not universal in their analytical capability, mainly due to limitations associated with chromatographic performance including “blind-spots” in the analysis for compounds eluting closely to major constituents (Herrington and Myers 2015). They do not easily identify very low molecular weight compounds, high boiling point species, nitro compounds, metals, most organic acids, tobacco-specific nitrosamines and compounds that require derivatisation prior to analysis (Rawlinson et al., 2017). Using a non-chromatographic approach that sampled e-cigarette puffs directly into a secondary electrospray ionisation (ESI) high resolution mass spectrometer (SESI-HRMS) García-Gómez et al. (2016) identified 142 compounds in an e-cigarette aerosol. SESI-HRMS has challenges with detection of low molecular weight species and compounds that are not easily ionised by ESI (such as PAHs), compound identification, and quantification. All of the techniques employed to date have limitations, and none are capable by themselves of fully characterising the chemical composition of an e-cigarette aerosol.

Therefore, despite it being a fundamental step in understanding e-cigarette science, and central to current public health concerns over vaping risks, there remains ongoing uncertainty concerning the chemical composition of e-cigarette aerosols. This is a basic characteristic defining e-cigarette aerosol properties, serving as a gateway to more complete studies of their chemical toxicity. Our study seeks to address this gap by combining untargeted and targeted analytical methods to more completely characterise the chemical composition of e-cigarette aerosols. We used the untargeted GC-MS method described by Rawlinson et al. (2017) and 18 additional targeted validated chemical assays for 98 specific compounds. The targeted methods covered many compounds that are poorly dealt with by the untargeted scan, including metals, nitrosamines, permanent gases, low molecular weight compounds, compounds requiring derivatisation and high boiling point aromatic species such as PAHs and aromatic amines. We examined aerosols from three common examples of e-cigarette flavors, tobacco, mint and a fruit flavor, in the same e-cigarette. This analytical strategy provided some insights into the impact of flavor complexity on aerosol composition. We further used quantitative data to conduct a mass-balance of the aerosol composition, providing some insights into the proportion of the aerosol made up by constituents other than the main e-liquid ingredients. Finally, these measurements were conducted in comparison to the mainstream smoke from a reference tobacco cigarette.

## Experimental Procedures

### Products

The e-cigarette used in this study, Vype ePen2 (Nicoventures Trading Ltd., Blackburn, United Kingdom), was an updated version of Vype ePen (as tested by Margham et al., 2016). Like the earlier version, it consisted of a rechargeable battery section and a disposable e-liquid containing cartridge (eCap). The battery section comprised a USB-rechargeable battery and an integrated circuit power controller with two voltage settings selectable by the consumer *via* an external twin-setting surface mounted switch. Device operation commences when the user presses either setting of the power switch, usually in advance of the puff starting, with power operating within the device as long as the button remained pressed. The liquid contained in the eCap was fed to the atomizer through a sintered porous ceramic disk in contact with a silica transport wick. The atomizer comprised a 2.85 *Ω* nichrome (80% Ni/20% Cr) wire coil heater wrapped around the wick. The updates incorporated into the ePen2 model included physical alterations to the dimensions and appearance of the device and cartridge, and also changes to the electronic features such as micro-USB charging and a reduction in the voltage settings from 3.6–4.0 to 3.5–3.7 V range for the low and high power settings, respectively.

The e-liquids studied were contained in Vype eCaps, that are disposable e-liquid cartridges containing 1.58 ml of e-liquid. In this study, we tested Golden Tobacco (ePen2GT), Dark Cherry (ePen2DC), and Crisp Mint (ePen2CM) flavored e-liquids. All the liquids contained VG, PG, water, flavors, and nicotine. The ingredient specifications for the e-liquids used in this study are shown in Table 1. The ingredients used in the three flavors were toxicologically assessed using the approach of Costigan and Meredith (2015). Also shown in Table 1 are the specifications for a non-commercial, unflavored e-liquid (referred to as “ePen2NF”) that we included in our untargeted aerosol emissions investigations. All the e-liquids had similar specifications (w/w) for water (25.00%) and nicotine (1.86%). The DC, GT, and unflavored e-liquids had the same specifications for VG (48.14%) and similar specifications for PG (23.86–25.00%), while the specifications for the CM e-liquid were 37.64% for VG and 34.73% for PG. The total percentage incorporation of all flavor compounds (the sum of all individual compounds) in the e-liquids was 0.77% for CM, 1.14% for DC, and 0.03% for GT. Commercial manufactured e-cigarettes from a single batch were tested (for both device and eCaps). Quality control checks were conducted on physical characteristics of all products against their manufacturing specifications before conducting the chemical analyses. This e-cigarette has an operating life of over 200 puffs per cartridge, depending on usage patterns, and we conducted these tests at the “High Power” setting (3.7 V).

**TABLE 1:** Specified composition of the e-liquids used in this study.

eCap flavor	Product code	Proportions of ingredients (% w/w)				
		VG	PG	Water	Nicotine	Other (flavors)
CM 18 mg/ml	ePen2CM	37.64	34.73	25.00	1.86	0.77
DC 18 mg/ml	ePen2DC	48.14	23.86	25.00	1.86	1.14
GT 18 mg/ml	ePen2GT	48.14	24.97	25.00	1.86	0.03
Unflavored	ePen2NF	48.14	25.00	25.00	1.86	0.00

The tobacco cigarette used for comparison in the current work was the Ky3R4F Kentucky Reference Cigarette, a US-blended king-sized product that has been widely used as a standard test-piece for scientific studies. It has a cellulose acetate filter and a tar yield under ISO 3308 puffing conditions International Organization for Standardization, (2012) of 9.4 mg/cigarette in 9 puffs. Main technical specifications are available on the website of the Kentucky Tobacco Research and Development Center, (2017). The mainstream smoke HPHC yields of the Ky3R4F under both ISO 3308 and ISO-Intense smoking conditions (ISO 20778:2018) have been reported previously (Roemer et al., 2012).

### Aerosol and Smoke Generation

Untargeted analyses were conducted at British American Tobacco R&D laboratories (Southampton, United Kingdom). Targeted analyses were conducted by Labstat International ULC (Kitchener, ON, Canada) using established methods developed, validated, and operated according to ISO17025. In all cases, aerosol, cigarette smoke, or Air/Method Blanks (AMBs) were generated using commercial puffing machines, adapted where necessary to accommodate e-cigarette button activation as part of the puffing cycle.

For untargeted analyses 80 ml puffs, over 3 s, taken twice per minute were produced using a Borgwaldt LX1 automated syringe unit (Borgwaldt KC, Hamburg, Germany), and the generated aerosol collected on conditioned Tenax TA/Sulficarb thermal desorption tubes (Markes International, Llantrisant, United Kingdom). For targeted emission testing, the e-cigarette puffing regime used was the Recommended Method 81 developed by the Cooperation Centre for Scientific Research Relative to Tobacco (CORESTA) for machine puffing of e-cigarettes.

Both of these methods reflect the longer puff duration commonly observed (McAdam et al., 2019) for e-cigarette users as compared with cigarette smokers (CORESTA 2015). The e-cigarette puffing regime specifies a puff volume of 55 ml, and a puff duration of 3 s, taken twice per minute (ISO 20768, 2018a). For products that require button activation to initiate aerosol generation, CORESTA specifies the activation timing parameters. In the current study, the activation button was pressed 1 s before each puff and held down for the duration of the puff (4 s in total for each puff). For this study, we activated the button using robotic, programmed devices synchronized to the puffing engines. Ky3R4F cigarettes were prepared for smoking according to ISO 3402 (International Organization for Standardization, 1999) and smoked under two different smoking regimes, the ISO 3308 International Organization for Standardization, (2012) and the ISO-Intense regimes (Health Canada 1999, ISO 20778, 2018b). The ISO 3308 regime specifies a 35 ml puff volume and 2 s puff duration taken once per minute. The ISO-Intense regime is an internationally standardized version of the smoking regime introduced in 1999 by Health Canada to compensate for potential blocking of the filter ventilation holes during smoking and to reflect the larger puff volumes taken by many smokers. The regime specifies a 55 ml puff volume and a 2 s puff duration taken twice per minute, with all filter ventilation holes blocked. The ISO-Intense smoking regime results in higher smoke yields than the ISO regime. When cigarettes are machine-smoked, the butt length and hence tobacco rod length smoked are predetermined. Under ISO 20768 puffing conditions, the e-cigarette cartridge provides more than 200 puffs of aerosol before the e-liquid becomes exhausted, and the Ky3R4F cigarette yields about 9 puffs under ISO 3308 conditions and 9–12 puffs under ISO-Intense parameters (ISO 20778:2018).

For targeted analyses, emissions data were collected on a per-cigarette basis for Ky3R4F, with the puff number recorded. For the e-cigarettes, the analyses were conducted on the cumulative emissions collected over 100 puffs. In the earlier paper by Margham et al. (2016), emissions were collected and analyzed from two successive 100 puff blocks. Since no significant differences were found between the first and second 100-puff block, it was decided to analyze only the first 100 puffs in the present study. The reported data for the Ky3R4F and the e-cigarette variants each comprises five independent replicates of products sampled at one point in time.

E-cigarette puffing and cigarette smoking were conducted in different dedicated laboratories, to minimise the potential for atmospheric contamination from cigarette smoke on e-cigarette measurements. AMB measurements were conducted to control for potential laboratory background levels of the target analytes (Margham et al., 2016; Wagner et al., 2018). For the e-cigarettes, AMBs were generated by drawing 100 puffs of laboratory air through empty ports of the puffing machine, and samples were analyzed in the same way as aerosol samples. For reference cigarettes, AMB measurements were also taken by drawing puffs through empty ports of the smoking machine under both ISO 3308 and ISO-Intense conditions.

### Chemical Analysis of Emissions From the E-Cigarette Aerosol and Cigarette Smoke

The untargeted aerosol scan was conducted using the semi-quantitative screening method described by Rawlinson et al. (2017). This method detects volatile and semi-volatile organic compounds with volatilities in the range from C_3_ hydrocarbons up to C_28_ hydrocarbons. PAHs with 5 or more rings, as well as other high molecular weight species with low volatilities at 250°C, are not detected by this method. The analysis is semi-quantitative, with compound concentrations estimated in comparison to known quantities of internal standard compounds. The method has been described in detail by Rawlinson et al. (2017) and is summarised in Supplementary File S1.

The emissions of major components (total aerosol and smoke masses, VG, PG, nicotine and water) from tobacco and electronic cigarettes were measured by trapping the generated smoke and aerosol on Cambridge Filter Pads (CFP). The total mass gained by the CFP during cigarette smoke experiments is defined as the TPM. With e-cigarettes, the same approach provides the gravimetric determination of ACM. Chemical analysis of TPM for nicotine and water allows for the calculation of the quantity (“nicotine-free dry particulate matter”) known as cigarette smoke tar. Chemical analysis of the ACM in this study for the major aerosol components PG, VG, water, and nicotine allows estimation of the mass of other unmeasured aerosol components. In both cases the quantity “Balance” was used, which was defined as the difference between either TPM or ACM and the sum of the major measured components.

A total of 98 individual compounds were measured in the emissions from the three flavor variants of the e-cigarette, the Ky3R4F reference cigarette, and respective AMBs. Many of these compounds are on one or more of the regulatory lists of harmful or potentially harmful cigarette smoke components. These include the Health Canada list of 42 toxicants in cigarette smoke (not including tar, nicotine and carbon monoxide) that are required to be measured and reported for cigarettes on the Canadian market (Health Canada 1999a), the FDA’s established list of 93 HPHCs in tobacco products and tobacco smoke (not including nicotine and carbon monoxide) of which 18 have to be reported currently (FDA 2012), and the World Health Organization list of 9 cigarette smoke components for which maximum levels have been proposed (Burns et al., 2008).

In analysing the 98 compounds of regulatory interest 18 different analytical methods were employed. Generally, groups of analytes belonging to the same chemical class were analyzed together in each method. Fewer compounds were tested than in the earlier paper by Margham et al. (2016) as that study showed no evidence for the presence of radioactive elements, polychlorinated dibenzodioxins or dibenzofurans in the e-cigarette aerosol. The concentrations of all these HPHC compounds were previously below the LODs of their measurement methods. The analytical methods used have been reported previously Margham et al. (2016), are summarised in Supplementary File S1 and described briefly here. Nicotine, propylene glycol, menthol, ethylene glycol, diethylene glycol, glycidol, and glycerol were analysed using GC/FID. Nicotine related alkaloids were analyzed by LC-MS/MS. Volatile carbonyls and dicarbonyls were determined by O-(2,3,4,5,6-pentafluorobenzyl)hydroxylamine (PFBHA) derivatization of trapped aerosol, followed by GC-MS analysis. Carbon monoxide was analyzed by non-dispersive infra-red analysis. Nitrogen oxides were analyzed using chemiluminescent techniques following reaction with ozone. Volatile and semi-volatile organic compounds were analyzed using GC-MS methods. Ammonia was analyzed by HPLC and conductivity detection. Hydrogen cyanide was quantified using continuous flow analysis. Phenolic compounds were analyzed by HPLC/FLD analysis. Aromatic amines were analyzed by GC-MS following derivatization by pentafluoropropionic acid anhydride. PAHs were quantified using GC-MS. Tobacco-specific nitrosamines were analyzed by LC-MS/MS. Volatile nitrosamines were analyzed by LC-MS. Metals were determined using inductively coupled plasma-mass spectrometry (ICP-MS) with a H_2_/He collision reaction interface.

### Data Analysis

#### Estimating Numbers of Aerosol Compounds

The chromatograms were interrogated using the following sequence to estimate the numbers of compounds detected in the aerosol:

A. The peaks in both aerosol samples and AMBs were attributed to specific compounds, the library Match Factor (MF) recorded, and numbers of peaks counted.
B. Duplicate compounds that eluted closely together in a chromatogram were counted as one peak, and the total peak number reduced accordingly.
C. Compounds that were present in the AMBs, at comparable levels to the e-cigarette aerosol levels, i.e., contaminants, were removed from the aerosol list.
D. The remainder constituted the total peaks in the non-targeted scan provided exclusively by the e-cigarette.
E. The number of compounds detected in the targeted analysis suite were counted; with the e-cigarette aerosols only those compounds present at levels > those in the AMB sample were counted.
F. Numbers of compounds detected in both untargeted (D) and targeted analyses (E), i.e. counted twice, were identified.
G. The totals from (F) were subtracted from the numbers counted in the targeted suite of analyses (E) to establish the numbers of compounds detected only in the targeted suite.
H. The totals of compounds found in the untargeted analysis (D) and only in the targeted analyses (G) were summed to provide the total number of compounds detected for each sample in this study.

Once the list of identified compounds had been assembled, we attempted to assign sources of these compounds in the e-cigarette aerosols, by categorising them into the following groups using knowledge of in-going materials and plausible reaction chemistries:

i. Known ingoing ingredients,
ii. Ingredient or device related minor constituents,
iii. Ingredient reaction products,
iv. Thermal decomposition products and
v. Compounds for which specific sources could not be assigned

With the unflavored ePen2 sample, which was not analysed for targeted analytes, typical targeted analytes found with the flavored samples were used for guidance purposes in this analysis.

#### Differences in Magnitude of Emissions From e-Cigarettes and Cigarette Smoke

Due to the substantial differences in puff numbers obtained from machine-smoking a tobacco cigarette (approximately 9–12 puffs) compared with an e-cigarette cartridge (up to several hundred puffs), the data are presented both “as measured” (i.e., per stick for Ky3R4F or per 100 puff block for the e-cigarette) and also on a per puff basis by dividing the reported values by the number of puffs taken during the measurement. The calculated per-puff values allow a direct comparison of emissions between products. Percentage differences between the emissions from the e-cigarettes and the Ky3R4F are calculated on a per puff basis.

We followed the same procedure as Margham et al. (2016) for calculating percentage differences in analyte concentrations from the e-cigarette aerosol that were below the limit of detection (LOD) or limit of quantification (LOQ) compared with smoke from the Ky3R4F cigarette results. For data < LOD, the value was calculated as one-half of the analytical method’s reported LOD. For data < LOQ but > LOD, the value was calculated as the midpoint between the reported LOD and LOQ of the analytical method:

Imputed value=reported LOD+0.5×(reported LOQ−reported LOD)

In cases where the e-cigarette and reference tobacco cigarette emissions were both < LOD or < LOQ, the measurand was omitted from the percentage difference calculations. In addition, the analysing laboratory provided “machine read values” when the test article measurement was >LOD but < LOQ. These enabled the comparisons to be conducted statistically in cases where a minimum of 3 replicates were reported > LOD but < LOQ.

Reductions in e-cigarette yields were calculated for each toxicant of regulatory interest (i.e., that appears on one or more lists) except where the yields were <LOD for both the e-cigarette and Ky3R4F cigarette. Where nicotine was on the toxicant list, composite yield reductions were calculated both with and without nicotine. Toxicant yields from the e-cigarettes were compared to yields from the Ky3R4F cigarette determined using the ISO-Intense smoking regime. The composite percentage reductions were calculated as the average reductions for all the toxicants on each list. Calculations were conducted without subtraction of AMB values.

Differences in results between products were tested for statistical significance (at the 95% level) using one-way analysis of variance (ANOVA) with a Tukey’s pairwise comparison, or two-way T-tests, where appropriate. Comparisons included toxicant concentrations in the e-cigarette aerosols vs. the AMB measurements, across the different flavored aerosols and between the e-cigarette aerosols and the smoke from the Ky3R4F cigarettes obtained under different smoking regimes. Data analyses were performed using SAS software (SAS System for Windows Version 9.4, SAS Institute Inc., Cary, NC, United States). Some additional analyses were carried out with the Minitab 16 statistical software package (State College, PA, United States).

## Results

### Aerosol Complexity

**FIGURE 1:**
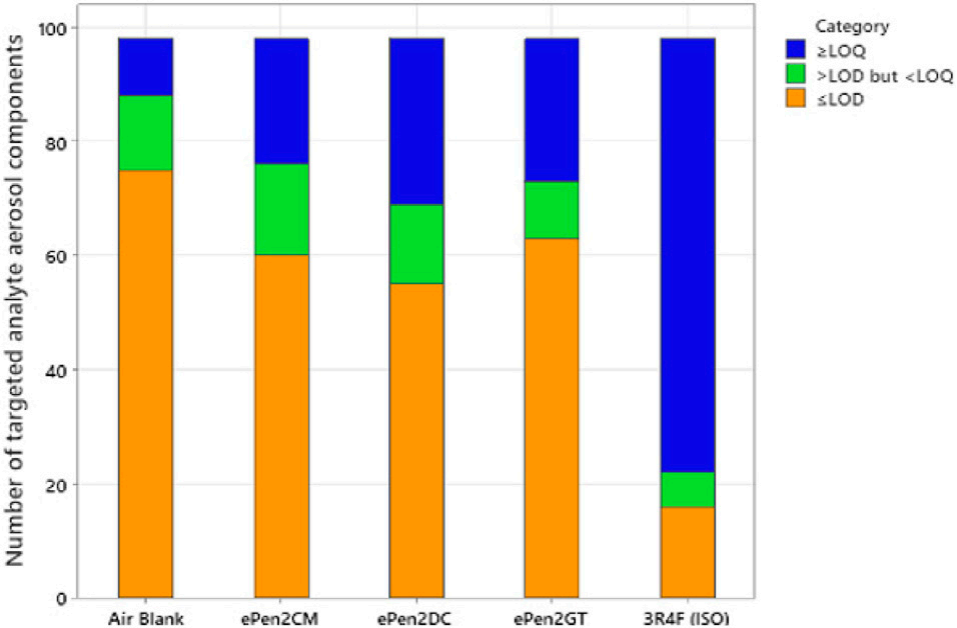
Number of targeted aerosol compounds in the three e-cigarette flavor variants, the air blank and the Ky3R4F cigarette (smoked under ISO conditions). The components are categorised according to whether their concentrations were quantifiable (>LOQ), detectable but not quantifiable (>LOD but <LOQ) or not detectable (<LOD).

Numbers of peaks quantified, detected but at levels too low to quantify, or undetected in the targeted analyses are presented in Table 2. These data are also illustrated graphically in Figure 1. Of the 98 compounds analysed, a total of 23 compounds were detected in the e-cigarette AMB. For the e-cigarette aerosols 35 (ePen2GT), 38 (ePen2CM), and 43 (ePen2DC) analytes were detected. For Ky3R4F smoke, under ISO 3308 smoking conditions, 82 were detected. In terms of quantifiable levels of the detected compounds, for the AMB 10 compounds could be quantified. For the e-cigarettes 22 (ePen2CM), 25 (ePen2GT) and 29 (ePen2DC) analytes were quantifiable. 31 of the targeted analytes were measured across all the samples (plus ACM) that could be quantified for at least one of the e-cigarettes. For Ky3R4F smoke 76 analytes were quantifiable.

**TABLE 2:** Numbers of the 98 targeted analytes undetected (≤LOD), detected but not quantified (>LOD but ≤ LOQ) and quantified (>LOQ) in the test articles and AMB.

Product	Number of targeted analytes reported			
	≤LOD	>LOD but ≤ LOQ	>LOQ	Total detected
ePen2CM	60	16	22	38
ePen2DC	55	14	29	43
ePen2GT	63	10	25	35
AMB	75	13	10	23
3R4F (ISO)	16	6	76	82

Findings from the untargeted analyses are summarised in Table 3. The AMB chromatogram contained 22 detected components (row B2), the unflavored e-cigarette (ePen2NF) aerosol had 68, and greater numbers of compounds were detected in the flavored e-cigarette aerosols: 89 for ePen2GT, 94 for ePen2CM, and 128 for ePen2DC. In each case a small number (0–3) were also detected in the AMB at comparable levels to the e-cigarette samples (although greater numbers of compounds were also detectable in the AMB at significantly lower levels than in the e-cigarette samples).

**TABLE 3:** Numbers of peaks detected in untargeted and targeted analyses.

	Air method blank (AMB)	ePen2NF	ePen2GT	ePen2CM	ePen2DC
A. Detected compounds in untargeted scan	22	72	91	99	131
B1. Number of duplicate peaks	0	4	2	5	3
B2. Detected compounds after subtraction of duplicates	22	68	89	94	128
C. Compounds in AMB also present at comparable levels to analysed ePen aerosol scan	-	1	0	3	1
D. Number of untargeted compounds generated exclusively by e-cigarette	-	67	89	91	127
E. Number of detected targeted analysis peaks (for the ePen samples only those > AMB were counted)	22	n/a	13	22	23
F. Compounds identified in both untargeted (D) and targeted analyses (E)	1	n/a	8	12	11
G. Compounds uniquely identified in targeted analyses	21	n/a (5–12)^a^	5	10	12
H. Total peaks detected (D + G)	43	>67 (72–79)	94	101	139

a Figure in parenthesis is an estimate for the number of compounds in the targeted scan of the unflavored e-cigarette aerosol; the estimate used was the range of targeted analytes detected with the flavored e-cigarettes.

Table 3 also shows further analysis of aerosol constituent numbers. The total numbers of aerosol constituents measured in this study were calculated for each aerosol (Table 3, row H); the total number of compounds from the untargeted analysis were added to those detected (whether quantifiable or not) in the targeted analyses. Those compounds identified in both targeted and untargeted analyses (8–12 for the flavored e-cigarettes) were counted once only. Table 3, row H shows that in total 43 compounds were detected in the AMBs. For the unflavored e-cigarette a total count was estimated because the targeted scans were not conducted on the unflavored variant; the range of values for compounds uniquely found in the targeted analyses of the flavored e-cigarettes suggested a range of 72–79 compounds in the unflavored e-cigarette aerosol. In contrast, greater numbers of compounds were observed in the three flavored e-cigarette aerosols, from 94 to 139, using the combined analytical techniques.

**FIGURE 2:**
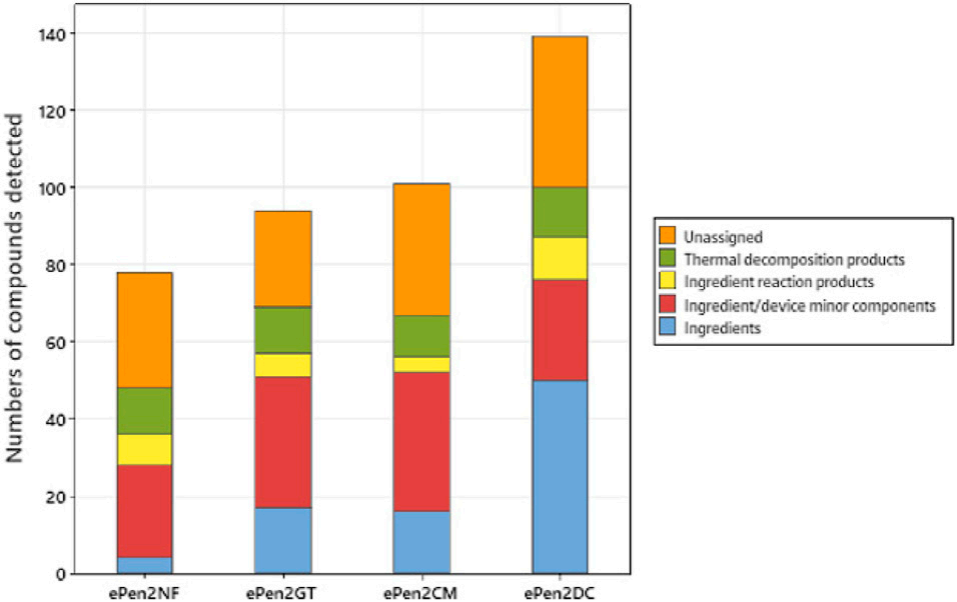
Assigned sources of aerosol compounds from the four e-cigarette variants. The components are assigned to ingredients, minor components of ingredients or device, reaction products, thermal decomposition products, and compounds whose sources could not be assigned.

The detected compounds were assigned to estimated sources as shown in Figure 2. Three e-liquid ingredients were detected in the aerosol of the unflavored e-cigarette. Had the targeted analyses been conducted on this sample water would have been detected, and hence number of ingredients would be four for this sample. With the flavored aerosols between 17 and 50 peaks were assigned as ingredients (with DC > CM > GT). Other sources of detected compounds in the aerosol included between 4 and 11 ingredient reaction products, such as acetals/hemiacetals/ketals formed by reaction of carbonyls with PG or VG. Compounds consistent with minor components of ingredients (e.g., minor components of flavors or solvent residues) or deriving from device materials (such as monomer residues) comprised 24 compounds (unflavored) and 26–36 compounds for the flavored aerosols. We also detected 11–13 compounds that were regarded as thermal decomposition products of ingoing ingredients. Subtraction of all these assigned compounds from the total numbers of compounds resulted in totals of 30 compounds for the unflavored aerosol and 25–39 compounds for the flavored aerosols that could not be assigned to a source. The inability to assign a source for these compounds was either because they could not be identified with sufficient confidence (e.g., low library match factor) or there was no clear explanation for their presence in the aerosol.

### Quantitative Analyses

In the targeted analyses the emissions of 98 aerosol components were quantified. The yields of these components for the three e-cigarette variants, the Ky3R4F cigarette (under both ISO and ISO-Intense smoking conditions), and the matching AMB samples are shown in Supplementary Table S1. As discussed above, 30 analytes were found in one or more of the e-cigarette aerosols and the emissions of these compounds are shown in Supplementary Table S2. These emission values are summarised in the following paragraphs:

#### Major Aerosol Components

**FIGURE 3:**
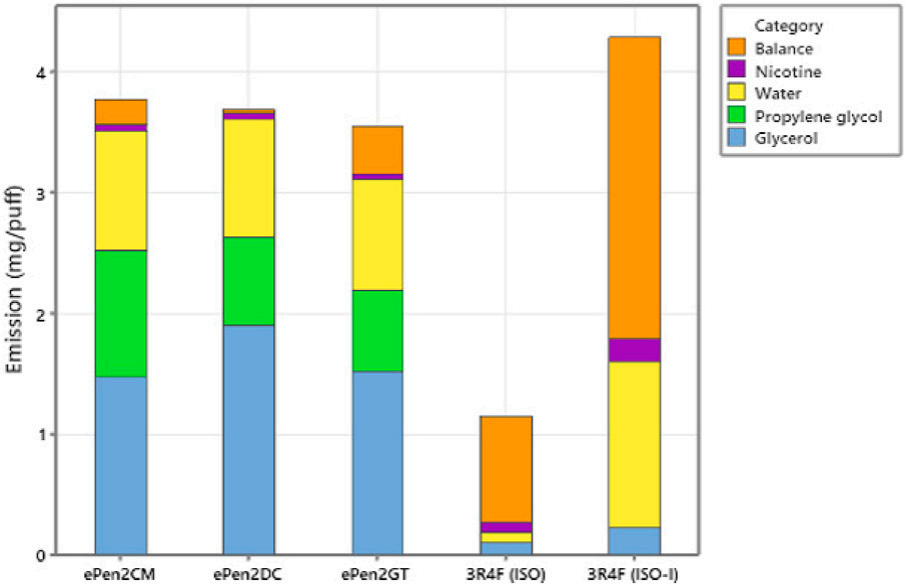
Major components of the aerosol masses collected from the three e-cigarette variants and the mainstream smoke from the Ky3R4F reference cigarette smoked under ISO and ISO-Intense regimes. The balance is the difference between the total collected mass and yields of glycerol, propylene glycol, nicotine and water. Emissions are in mg/puff.

ACM comprises the total mass of aerosol particles collected from the e-cigarettes generated during the puffing block. Table 4 and Figure 3 shows the major ACM components were the e-liquid compounds VG, PG, water, and nicotine. ACM values for the three e-cigarettes did not differ significantly. We note that the measured aerosol VG emission for GT was lower than expected by 0.3 mg/puff (by comparison to the other two products, factoring in their initial VG compositions and ACM/puff). Repeat analysis provided a higher per-puff VG in emissions, but higher than expected. We therefore reported the original measured value but note our concern over its robustness. There were differences in aerosol VG and PG concentrations between the e-cigarette variants consistent with the specified e-liquid compositions shown in Table 1. There were also differences in the values for “Balance” between the e-cigarette variants, with the GT variant having a higher value (11% of the ACM value—much of which can be explained by the VG measurement issue) than the CM (5.6% of the ACM) and DC (0.8% of the ACM) variants. For the Ky3R4F reference cigarette, the major components of the ISO 3308 TPM were VG, water, and nicotine, and under ISO-Intense conditions were water, VG, nicotine, and PG. The Balance after subtracting these components from the ISO 3308 TPM was 0.87 mg/puff (76.4% of the TPM), and 2.5 mg/puff (58% of the TPM) under ISO-Intense.

**TABLE 4:** Comparison of total quantities of cigarette smoke (3R4F) and aerosol (ePen2), and their major smoke/aerosol components.

Components (mg/puff)	ePen2CM	ePen2DC	ePen2GT	3R4F (ISO)	3R4F (ISO-I)
ACM/TPM	3.77 ± 0.41	3.69 ± 0.42	3.54 ± 0.52	1.14 ± 0.12	4.29 ± 0.25
Water	0.99 ± 0.11	0.98 ± 0.07	0.92 ± 0.10	0.086 ± 0.015	1.38 ± 0.13
Nicotine	0.052 ± 0.006	0.049 ± 0.006	0.047 ± 0.08	0.085 ± 0.014	0.185 ± 0.008
VG	1.47 ± 0.16	1.90 ± 0.09	1.52 ± 0.17	0.10 ± 0.005	0.22 ± 0.005
PG	1.05 ± 0.11	0.73 ± 0.05	0.67 ± 0.10	<LOQ^a^	0.002 ± 0.005
Balance	0.21	0.03	0.39	0.87	2.50
Balance (% of ACM/TPM)	5.6	0.8	11	76	58

<LOQ—below limit of quantification, i.e. not quantifiable, ACM–aerosol collected mass from the e-cigarettes; TPM–total particulate matter from the tobacco cigarette; Balance–TPM less water, nicotine, glycerol and propylene glycol; ISO-I–Iso-Intense; VG–Glycerol; PG–propylene glycol.

a LOQ = 0.001 mg/puff.

#### Carbonyls

Ten of the eighteen carbonyls analyzed were not detected in the aerosols of the three e-cigarettes: isobutyraldehyde, methyl ethyl ketone, 3-buten-2-one, n-butyraldehyde, crotonaldehyde, acetoin, 2,3-butanedione (diacetyl), 2,3-pentanedione (acetyl propionyl), 2,3-hexanedione and 2,3-heptanedione (Supplementary Table S1). The remaining eight carbonyls had quantifiable concentrations in the aerosols of all three flavor variants of the e-cigarette (Supplementary Table S2). These were formaldehyde, acetaldehyde, acetone, propionaldehyde, acrolein, glycolaldehyde, glyoxal and methylglyoxal. There were no significant differences (at 95%) in per puff yields with any of the quantified carbonyls between the three different flavor versions, other than propionaldehyde and acetone (Table 5) despite the differences of the in-going ingredients between the three variants (Table 1). Two of the carbonyls analyzed–formaldehyde and acetone–had quantifiable levels in the e-cigarette AMB. The AMB levels of formaldehyde represented 5.6–19% of the yields measured for the e-cigarette products, while the levels of acetone were 34–50% of the e-cigarette yields. On a per puff basis, quantified carbonyl emissions from the e-cigarette were, depending on the carbonyl, 68.6->99.9% lower than ISO 3308 smoke yields from the Ky3R4F cigarette and 88.4->99.9% lower than ISO-Intense Ky3R4F smoke yields (Supplementary Table S1 and Supplementary Table S2). For the Ky3R4F AMBs, six carbonyls were quantifiable under ISO 3308 and three under ISO-Intense puffing conditions. Formaldehyde (AMB value 12–24% of the mainstream emission values), acetaldehyde (<1%) and 2,3-butanedione (<5%) were identified in both puffing regimes AMBs. Acetone (3% of mainstream smoke levels), methyl ethyl ketone (4%), and crotonaldehyde (16%) were quantified under ISO 3308 puffing conditions.

**TABLE 5:** Means (per collection) and ANOVA results for analytes with at least one quantifiable result.

Analyte	CM		DC		GT		AMB		P
ACM, mg	377	A	369	A	354	A	0.00	B	<0.001
“Tar,” mg	273	A	266	A	258	A	0.18	B	<0.001
VG, mg	147	B	190	A	152	B	0.13	C	<0.001
PG mg	105	A	73.1	B	66.9	B	0.13	C	<0.001
Water, mg	98.5	A	97.6	A	91.7	A	0.30	B	<0.001
Nicotine, mg	5.18	A	4.88	A	4.65	A	0.015	B	<0.001
Menthol, mg	0.613	A	0.121	B	0.014	C	0.006	C	<0.001
Allyl alcohol, µg	1.06	B	4.08	A	0.64	B	0.03	B	<0.001
Formaldehyde, µg	59.0	A	17.5	A B	39.8	A B	3.32	B	0.018
Acetaldehyde, µg	18.0	A	7.71	A B	10.3	A B	0.91	B	0.026
Acetone, µg	6.15	A B	6.94	A	4.84	B	2.40	C	<0.001
Propionaldehyde, µg	6.01	A	4.94	A B	2.30	B C	0.12	C	<0.001
Acrolein, µg	19.2	A	12.3	A	12.4	A	0.230	B	0.002
Glycolaldehyde, µg	9.95	A	2.43	AB	8.16	AB	0.187	B	0.02
Glyoxal, µg	4.78	A	1.71	AB	2.97	AB	0.063	B	0.013
Methylglyoxal, µg	6.46	A	6.26	A	5.24	A	0.039	B	0.005
Nicotine-N-oxide, µg	1.89	B	4.37	A	1.60	B	0.44	C	<0.001
Cotinine, µg	0.855	B	1.37	B	5.64	A	0.14	B	<0.001
Myosmine, µg	0.815	B	1.95	B	6.23	A	0.22	B	<0.001
Nornicotine, µg	2.57	B	7.39	A	2.52	B	0.12	C	<0.001
*β*-Nicotyrine, µg	0.658	B	2.20	A	0.095	C	0.095	C	<0.001
Chromium, ng	37.6	A	41.8	A	33.3	A	42.3	A	0.227
Iron, ng	26.5	A	95.6	A	14.3	A	22.6	A	0.222
Zinc, ng	52.9	A	96.5	A	44.1	A	38.1	A	0.119
Naphthalene, ng	16.9	A	15.4	A	19.6	A	15.9	A	0.596
Benzo(a)anthracene, ng	0.92	A	0.79	A	1.23	A	1.00	A	0.333
Chrysene, ng	2.40	A	2.38	A	2.98	A	2.75	A	0.445
Propylene oxide, µg	338	B	808	A	182	B C	78	C	<0.001
2-aminonaphthalene, ng	0.106	A	0.187	A	0.201	A	0.153	A	0.625
3-aminobiphenyl, ng	0.035	A	0.059	A	0.070	A	0.043	A	0.471
4-aminobiphenyl, ng	0.028	A	0.031	A	0.067	A	0.030	A	0.300
o-toluidine, ng	0.923	A	0.995	A	1.037	A	0.823	A	0.733
Ammonia, µg	9.30	A	9.25	A	7.70	A	6.02	A	0.241

The use of the letters A, B, C and D in the table indicates whether the differences in the mean values between products or AMB are statistically significant or not. For a particular analyte, results for products that share the same letter are not significantly different, and where the letters differ the means are significantly different.

#### Phenolics

None of the phenolic toxicants measured were quantified in any of the e-cigarettes or the AMB. In contrast, all of these compounds were quantifiable (except resorcinol under ISO 3308 conditions) in mainstream smoke from Ky3R4F under both puffing regimes. None of the compounds were detected in the cigarette smoke AMBs. Emissions from the e-cigarettes were >99% lower than in cigarette smoke.

#### Gases and Volatiles

Of the 12 gases and volatiles analyzed, only ammonia was quantified in all three of the e-cigarette variants and was the only gas quantified in the e-cigarette AMB. There were no significant differences in yields between any of the e-cigarette products or corresponding AMB (Table 5), and its presence was attributed to AMB contamination. In contrast, the cigarette AMB did not have quantifiable levels of ammonia. E-cigarette ammonia emissions were 91–93% lower (ISO 3308) and 97% lower (ISO-Intense) than from Ky3R4F. Propylene oxide was quantified in the DC e-cigarette and was <LOQ from the other 2 variants. Propylene oxide was not detected in either of the cigarette or e-cigarette AMBs. Compared with yields from the Ky3R4F cigarette, e-cigarette PO emissions were >73% lower (ISO 3308) and >91% lower (ISO-Intense). Nitric oxide and hydrogen cyanide were each detected in one of the three e-cigarettes but not in the others. None of the other gases and volatiles - CO, NOx, 1,3-butadiene, isoprene, acrylonitrile, benzene, toluene, and ethylene oxide were detected in the aerosol or AMB, other than toluene being detected but not quantified in the AMB. The tobacco cigarette AMB had quantifiable levels of several compounds: 1,3-butadiene, isoprene, acrylonitrile, benzene, and toluene. The measured toluene in the AMB reached 8% of the mainstream smoke emissions, but levels of the other compounds were lower, at 3% and less of the respective mainstream smoke emissions.

#### Polycyclic Aromatic Hydrocarbons (PAHs)

All three of the e-cigarettes and the e-cigarette AMB had quantifiable levels of naphthalene and chrysene. AMB levels were not significantly different from the e-cigarette emissions (Table 5). The cigarette smoke samples contained substantially higher levels of these compounds, with the AMB values at up to 6% of the cigarette smoke value. Benzo(a)anthracene was quantified in the aerosol of GT and was detected but not quantified in the other two variants and AMB; e-cigarettes and AMB values were not significantly different. Emissions from Ky3R4F were substantially higher. The matching cigarette AMB level was not quantifiable under ISO 3308 conditions, but quantified at 6% of the smoke yield under ISO-Intense conditions. Benzo(a)pyrene was <LOQ in two of the e-cigarette aerosols, but not detected in the other e-cigarette aerosol or the AMB. Benzo(b)fluoranthene was <LOQ in two of the e-cigarettes and the AMB, but undetectable in the other variant. These two compounds were quantified in the AMB for the reference cigarette at up to 5% of the mainstream smoke emissions. Indeno(1,2,3-cd)pyrene and benzo(k)fluoranthene were not detectable for any of the e-cigarettes or the AMB. Where quantifiable, e-cigarette PAHs emissions were 98.4–99.6% (ISO 3308) and 99.1–99.8% (ISO-Intense) lower than from the Ky3R4F cigarette.

#### Aromatic Amines

Four of the eight aromatic amines analyzed were quantified in some of the e-cigarettes: 2-aminonaphthalene, 3- and 4-aminobiphenyls, and o-toluidine. Of these, all but 4-aminobiphenyl were also quantified in the e-cigarette AMB. Levels measured for e-cigarettes and AMB were not significantly different (Table 5). Ky3R4F mainstream smoke contained quantifiable levels of all of the measured aromatic amines except benzidine, which was not detected. Five of the aromatic amines were quantified in the reference cigarette AMB, but not consistently across puffing regimes. Levels were <1% of the mainstream smoke emissions, other than o-anisidine, which gave levels up to 13% of the Ky3R4F mainstream emissions. Reductions in the e-cigarette aromatic amine emissions compared with the ISO 3308 and ISO-Intense Ky3R4F yields were >99.6% in all cases.

#### Nicotine-Related Tobacco Alkaloids

Anatabine and anabasine were not detected in the e-cigarette aerosols or the AMB. Nornicotine, cotinine, and ß-nicotyrine (apart from GT) were quantified in the e-cigarette aerosols,. Myosmine was quantified in two of the e-cigarette aerosols (DC and GT) and detected in CM. Nicotine-N-oxide was quantified in one e-cigarette aerosol and detected in the other two variants. Other than nicotine and nicotine-N-oxide, levels of these compounds in aerosols from the e-cigarettes were >90% lower than in mainstream smoke from the reference cigarette. The e-cigarette AMB contained detectable levels of nicotine and cotinine but none of the other nicotine related alkaloids. None of these compounds were detected in the Ky3R4F AMB, other than nicotine, which was present at non-quantifiable levels.

#### Nitrosamines

There were no detectable levels of the four tobacco-specific nitrosamines (TSNA) or ten volatile nitrosamines in the e-cigarette aerosols or the AMB. All four of the TSNAs were quantified in the Ky3R4F smoke under both ISO 3308 and ISO-Intense conditions. Compared with the Ky3R4F smoke, reductions in the e-cigarette aerosol yields of TSNAs were >99.9%. Two of the volatile nitrosamines (N-nitrosopiperidine and N-nitrosopyrrolidine) were detected in Ky3R4F smoke (ISO regime) and AMBs. Their levels in the AMBs were a substantial proportion (50–66%) of the mainstream smoke emissions.

#### Metals

Mercury, cadmium, nickel, cobalt, beryllium, and tin were not detected in emissions from any of the e-cigarettes nor in the corresponding AMB. Ky3R4F emissions did not contain detectable levels of cobalt, beryllium, or tin. Nickel was detected (<LOQ) under both ISO 3308 conditions and ISO-Intense, but not in the corresponding AMBs. Both mercury and cadmium were quantified in Ky3R4F smoke but not detected in the corresponding AMBs. E-cigarette emissions of mercury and cadmium were 97->99% lower than in cigarette smoke.

Lead was detected but not quantified in all the e-cigarette emissions and the AMB. Lead was quantified in Ky3R4F mainstream smoke but not detected in the corresponding AMBs. E-cigarette emissions were >98% and >99% lower than from Ky3R4F smoked under the ISO 3308 and ISO-Intense regimes, respectively. Arsenic was detected but not quantified in all e-cigarette variants but was not detected in the AMB. Arsenic was quantified in cigarette smoke but not detected in the corresponding AMB. E-cigarette levels were therefore >85% and >93.8% lower than those from the Ky3R4F smoked under ISO 3308 and ISO-Intense conditions, respectively. Selenium and copper were detected but not quantified in the e-cigarette CM and DC variants and were not detected in the GT variant or in the AMB. Selenium was not quantifiable in Ky3R4F emissions, copper was quantified in cigarette smoke but not quantified in the corresponding AMB.

Iron and zinc were quantified in the DC variant but not quantified in the other e-cigarettes nor the AMB. Compared to Ky3R4F the levels from DC were 79% (iron) and 93% (zinc) lower (ISO 3308) and 74 and 96% lower respectively under ISO-Intense conditions. A comparison of Ky3R4F and its AMB data showed that substantial quantities of the measured iron and zinc in Ky3R4F mainstream smoke were found in the AMB (56–78% and 43–66%, respectively). Chromium was quantified in all the e-cigarette aerosols and the corresponding AMB, with e-cigarette values not significantly different to the AMB value. Emissions from Ky3R4F and corresponding AMBs were not quantifiable. Levels measured in both the e-cigarette aerosols and the corresponding AMB were 79–125% higher than the Ky3R4F ISO 3308 yield and 17–47% higher than the ISO-Intense Ky3R4F yield.

#### Semi-volatiles

Quinoline was not detected in the e-cigarette aerosols or the AMB. Pyridine and styrene were detected but not quantified in the DC aerosol and were not detected in the other e-cigarette aerosols or the AMB. All three compounds were quantified in Ky3R4F smoke and detected inconsistently at lower levels in the corresponding AMBs. E-cigarette levels of these compounds were >99% lower than from cigarette smoke.

#### Polyols and Alcohols

As noted previously, the humectants, VG and PG were the major constituents of the aerosols of the e-cigarettes. Allyl alcohol, a possible decomposition product of VG, was quantified in the aerosols of all three e-cigarettes. Ethylene glycol and diethylene glycol were not detected in any aerosol. Glycidol was <LOQ in one of the e-cigarette aerosols, and not detected in two variants and the AMB. Menthol, a flavor component, was quantified in the aerosols of the CM and DC variants of the e-cigarette, but was not quantifiable from GT. The Ky3R4F VG emissions were significantly lower than the yields from the e-cigarettes. None of the polyols or alcohols were quantified in the e-cigarette AMB except for PG. In the Ky3R4F cigarette AMB, allyl alcohol was detected under both puffing regimes at 4–8% of the cigarette mainstream smoke emissions.

### Semi-Quantitative Analyses

It is also of interest to understand the quantities of compounds present in the untargeted analysis. However, full quantitation was not achievable in the untargeted analysis because compound identities were not verified, and moreover where library match factors were low it is possible that the assigned identities were incorrect. Uncertainty over compound identity meant that it was not possible to conduct MS calibrations, which renders the concentration data semi-quantitative at best, providing “order of magnitude” information only. The estimated concentrations of detected compounds in aerosols of the e-cigarette variants that were not common to the AMB and disclosed ingredients were calculated. Of the aerosol components with Mass Spectral Library match factors (MF) ≥75, 65% had concentrations in the estimated range 0–20 ng/puff, and 36% had estimated levels of 0–5 ng/puff. Of the aerosol components with 50 ≤ MF < 75, 79% had estimated levels in the range 0–20 ng/puff, and 21% had estimated concentrations of 0–5 ng/puff. The highest estimated value of around 450 ng/puff was found for a compound eluting at 7.14 min in the chromatogram of the unflavored e-cigarette aerosol. The peak had a poor match factor to the MS library (70%); the best match was a silane–benzeneacetic acid, alpha, 4-bis [(trimethylsilyl)oxy-, trimethylsilyl ester. However, the poor match factor means that its identification should be regarded as tentative at best.

### Composite Reductions in Toxicants Compared to Cigarette Smoke

We calculated the composite average percent reductions in toxicant yields from the e-cigarettes compared with those from the Ky3R4F cigarette for different regulatory interest “lists” shown in Supplementary Table S3. Table 6 shows that the WHO TobReg 9 constituents were reduced by 98.5–99.5% in the emissions from all three flavor variants of the e-cigarette when compared on a puff-by-puff basis with smoke from a Ky3R4F cigarette smoked under the ISO-Intense regime. Although not statistically significant, the slightly lower percentage reduction for formaldehyde in CM (>88%) compared with the other flavor variants (96.6 and 92.2%) caused a slightly lower composite percentage reduction for the CM flavor variant. Toxicants on the FDA abbreviated list of 18 compounds generated under ISO-Intense were reduced on average by >97% compared with the Ky3R4F; the % reductions increased to >99% when nicotine is removed from the list.

**TABLE 6:** Composite percentage reductions in yields from the ePen2 variants vs. the 3R4F smoked under ISO-I for toxicants listed by WHO TobReg and Health Canada.

Toxicant list	Number of toxicants on the list	ePen2CM	ePen2DC	ePen2GT
		Composite average reduction per puff vs. 3R4F ISO-I (%)		
WHO TobReg Mandated list^a^	9	98.5	99.5	99.0
FDA abbreviated list	18	97.5 (99.0 excluding nicotine)	98.1 (99.5 excluding nicotine)	97.9 (99.3 excluding nicotine)

a 9 toxicants proposed by WHO TobReg to be mandated for lowering.

## Discussion

### Chemical Complexity of e-Cigarette Aerosols in Comparison to Cigarette Smoke

#### Chemical Complexity of e-Cigarette Aerosols

In this study, between 94 and 139 compounds were detected in the flavored e-cigarette aerosols, and an estimated 72–79 compounds in the aerosol from the unflavored e-cigarette aerosol. The differences between flavored and unflavored e-cigarettes reported here (Figure 2) demonstrate the contribution of flavor ingredients to the overall composition of e-cigarette aerosols. Havermans et al. (2021) reported the identification of an average of 10 ± 15 flavor compounds in their analysis of more than 100 e-liquids, whilst studies by a range of authors (Aszyk et al., 2018; Behar et al., 2018; Bitzer et al., 2018; Czoli et al., 2019; Hua et al., 2019; Hutzler et al., 2014; Lisko et al., 2015; Omaiye et al., 2019; Tierney et al., 2016) reported the detection of between 1 and 47 flavoring chemicals in individual e-liquids. Our findings of 15–67 additional compounds in the aerosols of flavored e-cigarettes compared to an unflavored sample are consistent with published values for flavor complexity of e-liquids, particularly when the potential for the presence of additional reaction products (such as acetals) between flavor compounds and PG is considered, as well contributions (Figure 2) to compound counts from minor components of ingoing ingredients (Bitzer et al., 2018).

Combining targeted and untargeted analyses clearly provides a more complete picture of aerosol complexity than untargeted analyses alone. In the present study a further 5–12 compounds were detected through use of the targeted analyses over and above those detected in the untargeted GC-MS analysis alone. However, our approach cannot be viewed as a complete characterisation of aerosol complexity, as the scope of the analyses was subject to three main limitations. First, the untargeted TD-GC-MS method used in this study adopted chromatographic heart-cutting to avoid detector overload by PG and VG; it is possible that aerosol components with similar retention times to the major constituents could be missed. Second, a particular weakness of the present study was a relatively limited examination of elemental species. In our study we analysed for thirteen metals but a broader range of elemental species can also be tested for, as demonstrated by Williams et al. (2013). In their study Williams et al. examined e-cigarette aerosols for the presence of 24 additional elements to those examined in this study. Four elements (Bi, Ir, Pd and Ti) were not detected from any e-cigarette, six were detected in only one sample (In, La, Mn, Rb, Ag and W), and several (Al, Ba, Ca, Mg, K, Na, Si, Sr and Zr) were found in most of the samples examined. In principle, the results of Williams et al. (2013) suggests that 6–20 elements may be present in the aerosols studied in the present work, in addition to the 72–139 compounds detected in this study. A third potential limitation of our study is that other compounds may exist within the aerosol that are incompatible with the untargeted GC-MS and targeted analytical methods. The combination of these three limitations means that our study is likely to have underestimated the total aerosol complexity of these e-cigarettes, but possibly not to a substantial degree.

The numbers of compounds reported here are generally higher than reported from studies using untargeted GC-MS analyses alone. For example, Rawlinson et al. (2017) detected 51–87 compounds in the aerosols from flavored second-generation modular e-cigarette devices, while Herrington et al. (2015) (Herrington and Myers 2015) detected 85 compounds in the aerosol from a flavored first-generation e-cigarette, 8 of which were common to the AMB. The other study in which untargeted e-cigarette aerosol analysis was reported (García-Gómez et al., 2016) did not use GC-MS, but rather employed direct sampling secondary electrospray ionization-high resolution mass spectrometry (SESI-HRMS) and detected comparable numbers of compounds (142) to one of the e-cigarettes detected in this study. The higher compound count reported by (García-Gómez et al., 2016) than Herrington et al. (2015) (Herrington and Myers 2015) and Rawlinson et al. (2017) may possibly reflect differences in the complexities of the e-cigarette aerosols examined by the various studies, alternatively it may reflect superiority of non-chromatographic ESI-HRMS for these purposes.

It is of interest to further understand the sources of detected compounds in the aerosols tested in this study. Assignment of sources is heavily dependent upon correct identification of compounds, and compound identification in the untargeted analysis used in the present study should be regarded as indicative, as they relied upon MS library matches. Further confirmatory steps, such as retention time matching, would be required to render the identities definitive. However, focusing on compounds with the highest MS library match factors suggested that the detected compounds were present in the aerosols due to a number of different sources.

Figure 2 shows that many of the compounds were ingredient related, whether aerosol former, nicotinic or flavor compounds. Reaction products of PG and VG such as acetals/hemiacetals and ketals further increased the contribution of ingredients to the compound count. Flavor compounds in particular had a significant impact on the numbers of detected compounds. The dependence of detected aerosol compound count on such ingredients, means that there is no simple fixed value for the numbers of compounds in an e-cigarette aerosol. Across manufacturers and products, flavor formulations can differ significantly in their compositional complexity, and the incorporation of natural flavor extracts (as opposed to synthetic flavor chemicals) will further drive complexity as extracts can offer substantial intrinsic compositional complexity. Furthermore, differences in device operating conditions across different products, notably power/temperature/time, could also be expected to impact the degree of e-liquid reaction or breakdown, thereby influencing aerosol complexity.

#### Comparison With Cigarette Smoke

Studies characterising the complexity of cigarette smoke indicate a substantially more diverse chemical environment than found with e-cigarette aerosols. Rodgman & Perfetti’s monograph on the composition of tobacco and tobacco smoke (Rodgman and Perfetti 2013) lists a total of over 6,500 identified tobacco smoke components. These include, of course, many compounds that would not be detectable with the analytical techniques used in this study. In the present work we were unable to conduct untargeted analysis on cigarette smoke with the available method due to the low capacity of the thermal desorption tube used in the analyses. However, a greater number of the targeted analytes (81) were found with cigarette smoke than with the flavored e-cigarettes (35–42).

Two studies have reported untargeted analysis of cigarette smoke. Brokl et al. (2014) conducted a scan of cigarette smoke’s particulate phase (but not the vapour phase) using headspace solid-phase microextraction coupled with 2-D GC-TOFMS, and detected >2000 GC-amenable compounds. Their findings point to cigarette smoke being 1-2 orders of magnitude more complex than the e-cigarette aerosols in this current study. A less sensitive scanning approach was reported by RJReynolds in a semi-quantitative gas chromatography study of smoke from a Kentucky Reference 1R4F cigarette (Reynolds, 1988). Their method, which was designed to detect compounds at >50 ng/puff, identified more than 660 compounds in cigarette smoke. In the current study between 12 and 19 compounds with yields ≥50 ng/puff, were found with the flavored e-cigarettes using both quantitative data from targeted analyses and semi-quantitative estimates of aerosol yield from the untargeted analysis. These counts were, again, 1-2 orders of magnitude lower than those found with in cigarette smoke.

### Quantified Emissions

Figure 3 shows that the sum of measured major e-liquid and aerosol constituents (VG, PG, water and nicotine) accounted on average for 94.2% of the ACM, (rising to 97% using the expected VG value for GT). The calculated difference between ACM and the sum of the major components (“balance”) is clearly sensitive to errors in the determination of the major species. Accurate quantification of water in aerosol streams has traditionally presented significant challenges and may also be associated with relatively large quantification errors in these measurements. In contrast, the comparable balance for cigarette smoke lay between 58 and 76% of the trapped particulate mass. These data also suggest a much more diverse composition of cigarette smoke compared to e-cigarette aerosols.

The carbonyl yields measured in the present study were not significantly different from those found previously by Margham et al. (2016), but the AMB values for formaldehyde, acetone, and MEK were significantly lower in the present study. Such compounds (formaldehyde, acetaldehyde, acetone, propionaldehyde, acrolein, glycolaldehyde, glyoxal, and methylglyoxal), and the compounds propylene oxide and allyl alcohol are thermal decomposition products of the humectants, PG and VG, used as aerosol generators (Stein et al., 1983; Laino et al., 2011; Laino et al., 2012; Sleiman et al., 2016).

Higher levels of aerosol nicotine and tobacco alkaloids were reported in the present study than by Margham et al. (2016) even though the e-liquid nicotine levels were the same in the two studies and ACM emissions were comparable. The tobacco alkaloids other than nicotine are present as low-level constituents of the pharmaceutical nicotine used in e-liquid formulations. Those quantified in the aerosol–nornicotine, myosmine, nicotine-N-oxide, cotinine, and ß-nicotyrine–are naturally present in the tobacco leaf used to produce the pharmaceutical grade nicotine used in e-liquids and some may also be formed through nicotine oxidation in e-liquids (Marion 1950; Kisaki et al., 1978; Martinez et al., 2014).

A number of metals were measured in the e-cigarette emissions. In an earlier paper (Margham et al., 2016) describing the aerosol chemistry of a similar product to that reported in the present study, chromium was quantified in the first 100 of 200 puffs at an average of 0.50 ng/puff but was not quantified (<0.45 ng/puff) in the second 100 puffs. The AMB contained detectable but not quantifiable levels of chromium (>0.13 but <0.45 ng/puff). Some levels of chromium generated by the e-cigarette could therefore not be ruled out. In the present study, levels of iron, zinc, and chromium found in the e-cigarette aerosols and the AMB were not significantly different (Table 5), and we can conclude that the presence of these metals likely arise as artifacts from aerosol collection or other analytical processes. For iron and zinc, Margham et al. (2016) also concluded that their presence in the aerosols was due to laboratory contamination. Williams et al. (2013) using non-standard smoking parameters analyzed 20 metals in the aerosol of a single brand of e-cigarette, including chromium (0.7 ng/puff), iron (52 µg/puff) and zinc (5.8 ng/puff), but none were detected in an AMB. Their results are significantly higher than those of the present study. Tayyarah and Long (2014) reported detectable but non-quantifiable levels of chromium (1–4 ng/puff) in three products they tested as well as the AMB. However, other studies, such as that of Goniewicz et al. (2014) have not detected chromium in e-cigarette emissions.

In the present study, consistent with the findings of Margham et al. (2016), emissions of four aromatic amines were quantified in the e-cigarette aerosols and AMB, with levels not significantly different between the background and aerosol samples. Three PAHs and ammonia were quantified in the e-cigarette emissions and AMB; levels were not significantly different between samples. Emissions of ammonia, chrysene, and naphthalene were higher in the present study for all the e-cigarettes as well as the AMB than found previously by Margham et al. (2016). TSNAs were not detected in any of the e-cigarette samples, even with picogram per puff LODs.

### Contribution From Laboratory Air, Analytical Equipment or Analytical Reagents

Given the very low levels of many of the toxicants that are now measured in e-cigarette aerosols, combined with the relatively large numbers of puffs taken on e-cigarettes in comparison to tobacco cigarettes, it is essential to understand the contribution to measured values from environmental factors. These include toxicants that may already be present in the laboratory air, in reagents, or that may be introduced by operators or equipment (such as puffing machines) used to generate and collect the aerosol. Hence the importance of the AMB as a means of minimising the possibility of false-positives and overestimates (Tayyarah and Long, 2014; Margham et al., 2016; Wagner et al., 2018; Belushkin et al., 2020). AMB control measurements are widely used in different e-cigarette research areas, such as chemical analysis and indoor air quality studies (Goniewicz et al., 2014; Herrington and Myers, 2015; Marco and Grimault, 2015; Mikheev et al., 2016; Palazzolo et al., 2016; Aherrera et al., 2017; Beauval et al., 2017; Moldoveanu et al., 2017; Olmedo et al., 2018; Halstead et al., 2019).

Table 5 shows that in the present study, there were eight components where AMB values were numerically higher than one or more of the e-cigarette aerosol samples. These were chromium (higher than all 3 e-cigarette samples), iron (higher than 1 sample), naphthalene (1), benzo(a)anthracene (2), chrysene (2), 2-aminonaphthalene (1), 3- and 4-aminobiphenyls (1 sample each). However, ANOVA testing showed that none of these differences were significant at the 95% confidence level. Therefore, in none of these cases was there a significant difference between AMB and the e-cigarette samples. In addition, the ANOVA tests showed that zinc, o-toluidine and ammonia emissions were not significantly different from those found in the AMB (Table 5).

Further insights into the potential presence or concentrations of these toxicants will require greater reductions of chemical background than are currently achievable using established methods. Gaseous and volatile contaminants could be excluded during e-cigarette experiments by use of air-tight assemblies fed by high purity air, as reported by García-Gómez et al. (2016). However, not all of the contamination arises from the laboratory air. Metals such as chromium, iron and zinc appear to arise at least in part from the puffing machines used to generate aerosols and the associated trapping matrices (data not shown); reducing the impact of metal contamination from these sources may represent a way to minimise AMB contamination with these compounds. These approaches represent valuable avenues for future investigation.

AMB experiments for the Ky3R4F cigarette were conducted under two puffing regimes, and contributions to the measured cigarette smoke emissions were found with 29 of the 98 analytes measured in this study. Levels per puff were generally much higher than found with the e-cigarette AMB, due in the main to some elements of environmental tobacco smoke around the smoking engine (generated by the cigarette sidestream smoke as it leaves the burning cigarettes) being pulled into the empty port of the smoking engine during the puffing steps of the AMB experiment. This source does not exist for the e-cigarette AMB experiment. Despite the higher absolute levels measured with the Ky3R4F AMB, their contribution to the measured levels in smoke was generally less due to the relatively high concentrations of toxicants in mainstream cigarette smoke.

## Conclusion

This study has demonstrated that e-cigarette aerosols contain significantly fewer toxic components and at lower concentrations than a reference cigarette. In contrast to the thousands of identified compounds in cigarette smoke, between 94 and 139 aerosol compounds were detected from flavored e-cigarettes when data from both targeted and untargeted analytical methods were combined. Using a combined approach provided greater compositional insights than either targeted or untargeted approaches alone. Identities of the detected e-cigarette aerosol constituents were attributed to sources including ingredients such as flavor compounds, reaction products of those ingredients, minor components of device and ingredients, thermal decomposition products, and compounds that could not be accurately identified.

Toxicant yields per puff from the e-cigarettes were 68–>99.9% lower than those from the reference cigarette under both ISO and ISO-Intense puffing conditions. Overall, the levels of the 9 WHO TobReg prioritized toxicants were around 99% lower than measured from the reference cigarette under ISO-Intense puffing conditions. Our results agree with the emerging scientific literature in that the e-cigarette aerosols are chemically much simpler than cigarette smoke, and contain fewer toxicants at lower concentrations.

## Data Availability

The original contributions presented in the study are included in the article/Supplementary Material, further inquiries can be directed to the corresponding author.

## Abbreviations

ACM, Aerosol Collected Mass (total weight of aerosol particulate matter from an e-cigarette, equivalent to TPM from a tobacco cigarette); AMB, Air/method blank; ANOVA, Analysis of variance; CM/ePen2CM, “Crisp Mint” flavored variant of the e-cigarette; CORESTA, Cooperation Centre for Scientific Research Relative to Tobacco; DC/ePen2DC, “Dark Cherry” flavored variant of the e-cigarette; FDA, US Food and Drug Administration; FID, Flame ionisation detector; GC/MS, Gas chromatography-mass spectroscopy; GT/ePen2GT, “Golden Tobacco” flavored variant of the e-cigarette; HPHC, Harmful and potentially harmful constituents; ICP-MS, Inductively coupled plasma-mass spectrometer; ISO, International organization for standardization; ISO-INTENSE (ISO-I), More intensive cigarette smoking regime than traditional ISO puffing parameters; LOD, Limit of detection; LOQ, Limit of quantification; MF, Match factor; NF, Non-flavored - an unflavored variant of the e-cigarette; PAH, Polycyclic Aromatic Hydrocarbon; TOFMS, Time-of-flight mass spectrometer; TPM, Total Particulate Matter (total weight of mainstream smoke from a cigarette); TSNAs, Tobacco specific nitrosamines.
