# Supplementary material for: The Chemical Complexity of e-Cigarette Aerosols Compared With the Smoke From a Tobacco Burning Cigarette
Source: Front Chem. 2021 Sep 30;9:743060. doi: 10.3389/fchem.2021.743060 (PMC8514950; doi:10.3389/fchem.2021.743060)
Supplement: Supplementary file 1 [file DataSheet2.docx]

**Supplementary File 1: Analytical Methods Used for Chemical Characterisation of E-cigarette aerosols and cigarette smoke.**

**1. Non-targeted aerosol analysis**

The untargeted scan approach was conducted on e-cigarette aerosol samples, but not the reference cigarette smoke sample. The purpose was to obtain a broad characterization of e-cigarette aerosol composition. For this study, we used the semi-quantitative screening method described by Rawlinson et al. [2017]. This method detects volatile and semi-volatile organic compounds with volatilities in the range from C_3_ hydrocarbons (e.g., propane) up to C_28_ hydrocarbons. PAHs with 5 or more rings, as well as other high molecular weight species with low volatilities at 250°C, are not detected by this method. The analysis is semi-quantitative, with compound concentrations estimated in comparison to known quantities of internal standard compounds.

Briefly, conditioned thermal desorption (TD) tubes (Inert coated with Tenax TA/Sulficarb, Markes International, Llantrisant, UK) were used to collect single 3 sec, 80 ml puffs from the e-cigarette using an automated syringe unit (LX1, Borgwaldt KC, Hamburg, Germany). The tubes were loaded onto a TD100 thermal desorption unit (Markes International, Llantrisant, UK) and dried with helium. Internal standards (bromochloromethane, 1-bromo-4-fluorobenzene, chlorobenzene-D5, and 1,4-difluorobenzene) were added, and the contents desorbed at 290°C into a cold trap (UT-6SUL-2S, Markes International, Llantrisant, UK).

Analytes desorbed from the cold trap were split with 1:201 transferred to the GC system for separation and detection and 200:201 re-collected on the sampled TD tube. The sampling process was repeated two additional times on the re-collected portion of the sample to detect any artefactual production of aerosol constituents. Analytes were separated using a gas chromatograph (Agilent 7890B) fitted with an RTX-Stabilwax column (30 m x 0.25 mm ID x 0.5 μm), and a Deans Switch coupled to an ALMSCO bench time-of-flight mass spectrometer (TOFMS) and a flame ionization detector (FID). The TOFMS was operated in electron impact ionization mode (70eV) and acquired ions in the m/z range 29 to 350 at a frequency of 8 Hz. The GC oven was initially set at 40°C and held for two minutes, followed by a 15°C/min ramp until a final temperature of 250°C was reached and held for an additional 20 minutes. The total run time was 36 minutes. A heart-cutting sequence was applied to isolate specific segments of the chromatogram (i.e*.* those time windows in which propylene glycol, glycerol, and nicotine eluted) to avoid overloading the TOF detector with the bulk constituents. Six replicates of each sample were measured. These analyses were carried out at British American Tobacco R&D Centre (Southampton, UK).

The GC-MS data were analyzed for chromatographic features and their chemical identities using Genedata Expressionist version 9.0 (Genedata AG, Switzerland). In this automated system, chromatographic features are only reported if they contain at least 15 ion fragments. Compounds that have less than 15 ion fragments greater than mass 29 may be present but may not be reported. The minimum compound size possible for being reported is 47 atomic mass units. Proposed identifications are based on comparisons of the mass spectra of the de-convoluted peaks with National Institute of Standards and Technology (NIST) library spectra, and only features that were present in five of the six replicates are reported. The Genedata library match factors are based on a scale of 0 to 100. A match score (Match Factor MF) of < 75 was treated as a tentative identification with low confidence, and the proposed identification was likely incorrect. A peak with MF>75 was treated as a tentative match with higher confidence, and the proposed identification was more likely to be correct. Compounds with MF< 75 were not reviewed, and those with MF> 75 were reviewed. The identities of these compounds have not been included in this report. Concentrations of each compound/peak were calculated as the average of all the estimated concentrations from each identified internal standard and are reported on a per puff basis (mean of 6 puffs). The concentrations should only be considered as an order of magnitude guide since relative response factors were not measured.

**2. Targeted Chemical Analysis of E-cigarette Aerosols and Cigarette Smoke.**

The methods used by the analysis laboratory have been described previously [Margham et al. 2016] and are summarised below. The methods used were largely based on Health Canada methods for cigarette smoke analysis, with additional methods developed by Labstat for the other HPHCs and e-cigarette compounds of interest. The methods were adapted for use with e-cigarettes where necessary. The operation of the methods is accredited to ISO/IEC 17025:200573 for all reported constituents of mainstream tobacco smoke and e-cigarette aerosols.

Nicotine, propylene glycol, menthol, ethylene glycol, diethylene glycol, glycidol, and glycerol in smoke/aerosol were trapped using a Cambridge Filter Pad (CFP) backed by a liquid impinger, extracted using isopropanol (IPA) and analyzed using GC/FID. Nicotine related alkaloids (nornicotine, anatabine, anabasine, myosmine, nicotine-N-oxide, cotinine, and ß-Nicotyrine) were analyzed by extracting CFPs with ammonium acetate solution followed by LC-MS/MS. Allyl alcohol was determined using methanol extraction of CFP/impingers followed by GC/MS detection.

Volatile carbonyls and dicarbonyls (formaldehyde, acetaldehyde, acetone, propionaldehyde, acrolein, isobutyraldehyde, methyl ethyl ketone, 3-buten-2-one, n-butyraldehyde, crotonaldehyde, glycolaldehyde, acetoin, glyoxal, methylglyoxal, 2,3-butanedione, 2,3-pentanedione, 2,3-hexanedione, and 2,3-heptanedione) were determined by O-(2,3,4,5,6-pentafluorobenzyl)hydroxylamine (PFBHA) derivatization of trapped aerosol, followed by GC-MS analysis. This is a different technique to that used by Margham et al. (2016) and was used in the present study as it offers a greater number of analytes in the same analysis suite while offering comparable or superior analytical performance (Bao et al. 2014).

Carbon monoxide was analyzed by non-dispersive infra-red analysis. Nitrogen oxides were analyzed using chemiluminescent techniques following reaction with ozone. Volatile organic compounds (1,3-butadiene, isoprene, benzene, toluene, acrylonitrile, ethylene oxide, and propylene oxide) were analyzed using GC-MS. Semi-volatile organic compounds (pyridine, quinoline, and styrene) were analyzed by methanolic extraction of CFP/impingers followed by GC-MS analysis. Ammonia was analyzed by trapping using a CFP/impinger, extraction using a 0.1N sulphuric acid, and analysis by HPLC and conductivity detection. Hydrogen Cyanide was trapped by CFP/impinger, extracted using NaOH solution, and quantified using a continuous flow analyzer. Phenolic compounds (phenol, catechol, hydroquinone, resorcinol, and o-, m- and p-cresols) were analyzed by extraction of the CFP with acetic acid followed by HPLC analysis with detection by FLD.

Aromatic Amines (1- and 2-aminonapthalene, 3- and 4-aminobiphenyl, o-toluidine, o-anisidine, 2,6-dimethylaniline, and benzidine) were analyzed by HCl/DCM extraction of CFPs, basification and hexane extraction, derivatization by pentafluoropropionic acid anhydride, and clean-up through a florisil column before analysis by GC-MS. PAHs (naphthalene, benzo[a]anthracene, chrysene, benzo[a]pyrene, indeno(1,2,3-cd)pyrene, benzo(b)fluoranthene and benzo(k)fluoranthene) were trapped using a CFP, extracted in methanol, cleaned-up using a C18 cartridge and elution with Cyclohexane followed by GC-MS quantification.

Tobacco-specific nitrosamines (N'-nitrosonornicotine, 4-(methylnitrosamino)-1-(3-pyridyl)-1-butanone, N'-nitrosoanatabine and N'-nitrosoanabasine) were trapped using a CFP, extracted using a 100 mM ammonium acetate solution and analyzed by LC-MS/MS. Volatile nitrosamines (NDMA, NEMA, NDEA, NDiPA, NDPA, NDBA, NPIP, NPYR, NMOR, and NDELA) were analyzed by extraction of CFP and impinger using ammonium sulfamate /sulfuric acid buffer. The extract was acidified, treated with ammonium sulphate, and then subject to ChemElut® clean-up using ethyl formate:ethanol (98:2, v/v) as eluent. The extract is reconstituted in formic acid before LC-MS analysis.

Metals (mercury, cadmium, lead, chromium, nickel, arsenic, selenium, copper, cobalt, beryllium, zinc, tin, and iron) were determined using a modified inductively coupled plasma-mass spectrometer (ICP-MS) method with a collision reaction interface used with hydrogen and helium to remove interferences.
